# Numerical modelling of blood rheology and platelet activation through a stenosed left coronary artery bifurcation

**DOI:** 10.1371/journal.pone.0259196

**Published:** 2021-11-03

**Authors:** David G. Owen, Diana C. de Oliveira, Emma K. Neale, Duncan E. T. Shepherd, Daniel M. Espino

**Affiliations:** Department of Mechanical Engineering, University of Birmingham, Birmingham, United Kingdom; University of New South Wales, AUSTRALIA

## Abstract

Coronary bifurcations are prone to atherosclerotic plaque growth, experiencing regions of reduced wall shear stress (WSS) and increased platelet adhesion. This study compares effects across different rheological approaches on hemodynamics, combined with a shear stress exposure history model of platelets within a stenosed porcine bifurcation. Simulations used both single/multiphase blood models to determine which approach best predicts phenomena associated with atherosclerosis and atherothrombosis. A novel Lagrangian platelet tracking model was used to evaluate residence time and shear history of platelets indicating likely regions of thrombus formation. Results show a decrease in area of regions with pathologically low time-averaged WSS with the use of multiphase models, particularly in a stenotic bifurcation. Significant non-Newtonian effects were observed due to low-shear and varying hematocrit levels found on the outer walls of the bifurcation and distal to the stenosis. Platelet residence time increased 11% in the stenosed artery, with exposure times to low-shear sufficient for red blood cell aggregation (>1.5 s). increasing the risk of thrombosis. This shows stenotic artery hemodynamics are inherently non-Newtonian and multiphase, with variations in hematocrit (0.163–0.617) and elevated vorticity distal to stenosis (+15%) impairing the function of the endothelium via reduced time-averaged WSS regions, rheological properties and platelet activation/adhesion.

## Introduction

Cardiovascular diseases were responsible for an estimated 18.8 million deaths in 2017 [1], with heart disease being the leading cause of death in the USA in the same year [2]. Of particular relevance to cardiovascular health are the coronary arteries, two major blood vessels which bifurcate into multiple smaller branches. The coronary arteries have been associated with several clinical pathologies, including atherosclerotic plaques/lesions [3, 4], arterial stiffening [5] and increased thrombosis [6] (clot formation). Atherosclerosis is the development of fatty plaques within the artery wall, with their onset and progression associated with regions of low time-averaged wall shear stress [7] (TAWSS) occurring around the branching of the artery [8] or distal to regions of stenosis [9]. In addition to plaque rupture causing myocardial infarction or sudden death [2], the altered hemodynamics arising from inflammatory plaque growth also impacts the behaviour and activation of platelets; catalysing plaque/fibrin growth through increased platelet adhesion and increased risk of thrombus formation [10] (atherothrombosis), with a detailed review of these phenomena provided by Davi and Patrono [11].

Computational fluid dynamics (CFD) is a useful tool to study vascular pathologies and is capable of predicting the location and progression of coronary atherosclerotic plaques [12], growth/rupture of aneurysms [13] and microscopic thrombus growth [14]. The branching of the coronary arteries results in low-shear and stagnant/recirculation environments occurring on the outer walls [15], being common sites for plaque growth and hence stenosis to occur. The realistic simulation of blood flow in these regions is crucial for accurate hemodynamic predictions and hence the understanding of disease mechanisms. There are multiple fundamentally different approaches to blood rheology [16], all based on fitting constituent equations to differing sets of experimental viscometer data [17]. Blood is a multiphase fluid, comprised predominantly of a dilute suspension of red blood cells (RBC) within a plasma continuum [18]. Rheological properties depend on fluid shear rates, the concentration of RBC [19] (hematocrit) and crucially the aggregation of individual RBC into a rouleaux, which is a key factor in non-Newtonian behaviour. Rouleaux formation requires prolonged exposure to low-shear rates [20] (<50 s^-1^), and as is inherently a microscopic phenomenon [21]. As there are approximately 5×10^6^ RBC per mm^3^ of blood [22], it is impossible to simulate this phenomenon for large arteries with current computational power. Shear-rates in larger arteries are conventionally considered to be sufficiently high for rouleaux formation to be negligible, in which case a more simplistic approach is to assume blood to be a homogeneous single-phase fluid, with constant density and either a constant [23] (Newtonian) or a shear-thinning (non-Newtonian) viscosity [24]. However, as a low-shear environment is commonly associated with disease, the extent to which this assumption oversimplifies crucial behaviour is contended [25]. As macroscale multiphase models do not simulate individual RBC, representing the rheological effects of aggregation and rouleaux formation is an ongoing challenge. However, the recent 5-parameter Modified Krieger Model (MKM5), is based upon the Krieger model for suspensions and attempts to incorporate the effect of aggregation on the viscosity of blood [26].

Many of the studies that focus on the impact of different types of rheology in coronary artery hemodynamics employ healthy geometries, and assume blood as a single-phase fluid [27–29], with fewer studies assessing the effects of multiphase models in coronary hemodynamics [30, 31]. Despite significant evidence on the non-Newtonian effects occurring around the coronary bifurcation, and the importance of hematocrit on the properties of blood, to date there is no conclusive study comparing these effects on parameters associated with the progression of atherosclerosis, and the subsequent impact on atherothrombosis/platelet activation.

The present study aims to compare the impact of different rheological assumptions on a diseased left coronary bifurcation (at the level of the left anterior descending artery, LAD, and the left circumflex artery, LCx). In particular, the prediction of blood flow in the low-shear environment distal to the bifurcation/stenotic regions, including the aggregatory potential of RBC via low-shear residence time and variations in hematocrit. Furthermore, the effect of stenosis on the transport/activation of platelets will be considered for a multiphase model, using a novel Lagrangian platelet simulation to evaluate trajectories, residence times and level of activation. A total of four rheological models were considered: single-phase Newtonian (SN); single-phase Carreau (SC) multiphase Newtonian (MN); and multiphase MKM5.

## Methods

### Geometry

#### Coronary bifurcation

**Fig 1:**
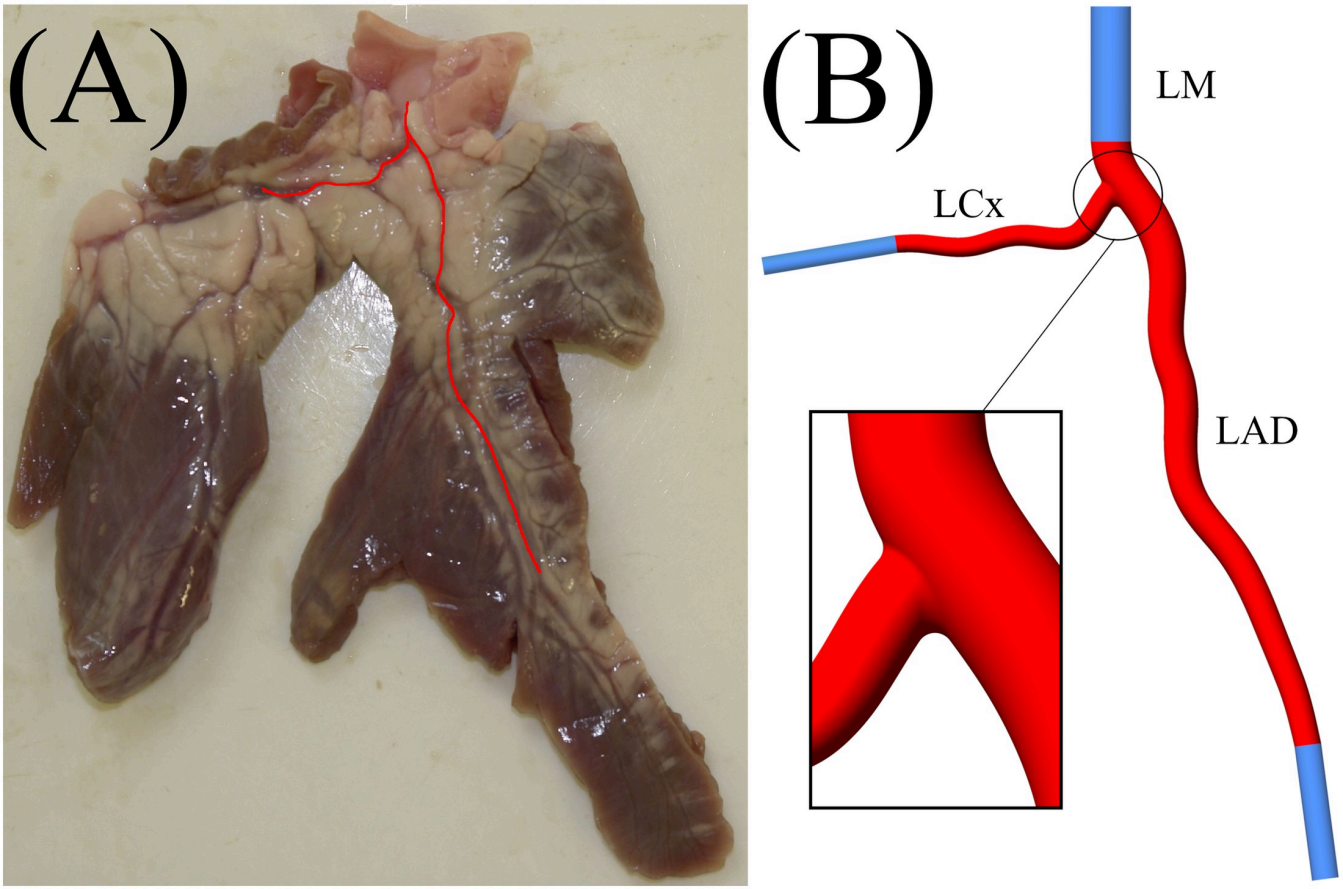
(A)—Porcine *ex vivo* heart segment, with coronary geometry marked in red. (B) Coronary artery bifurcation geometry including domain extensions shown in blue (LAD, left anterior descending artery; LCx, left circumflex artery; LM, left main coronary artery).

The vessel geometry was constructed using a centreline profile from an *ex vivo* sample of healthy porcine left main coronary artery bifurcation (LAD-LCx) from Fresh Tissue Supplies, Horsham, UK. This was scaled to match human physiological dimensions [32] using SolidWorks (SolidWorks, Dassault Systèmes, Vèlizy-Villacublay, France), and is shown in Fig 1. The total length of the artery is 70 mm, with a branch angle of approximately 72°, an inlet diameter of 4.5 mm, and outlet diameters of the LAD and LCx branch being 3 mm and 2 mm, respectively. The reduction in circular lumen diameter was achieved via linear-interpolation between the inlet-outlet resulting in a gradual tapering. To avoid any unwanted entrance/exit effects on the hemodynamics of the bifurcation, the inlets and outlets have been extended 15 mm using a constant diameter.

#### Diseased coronary bifurcation

**Fig 2:**
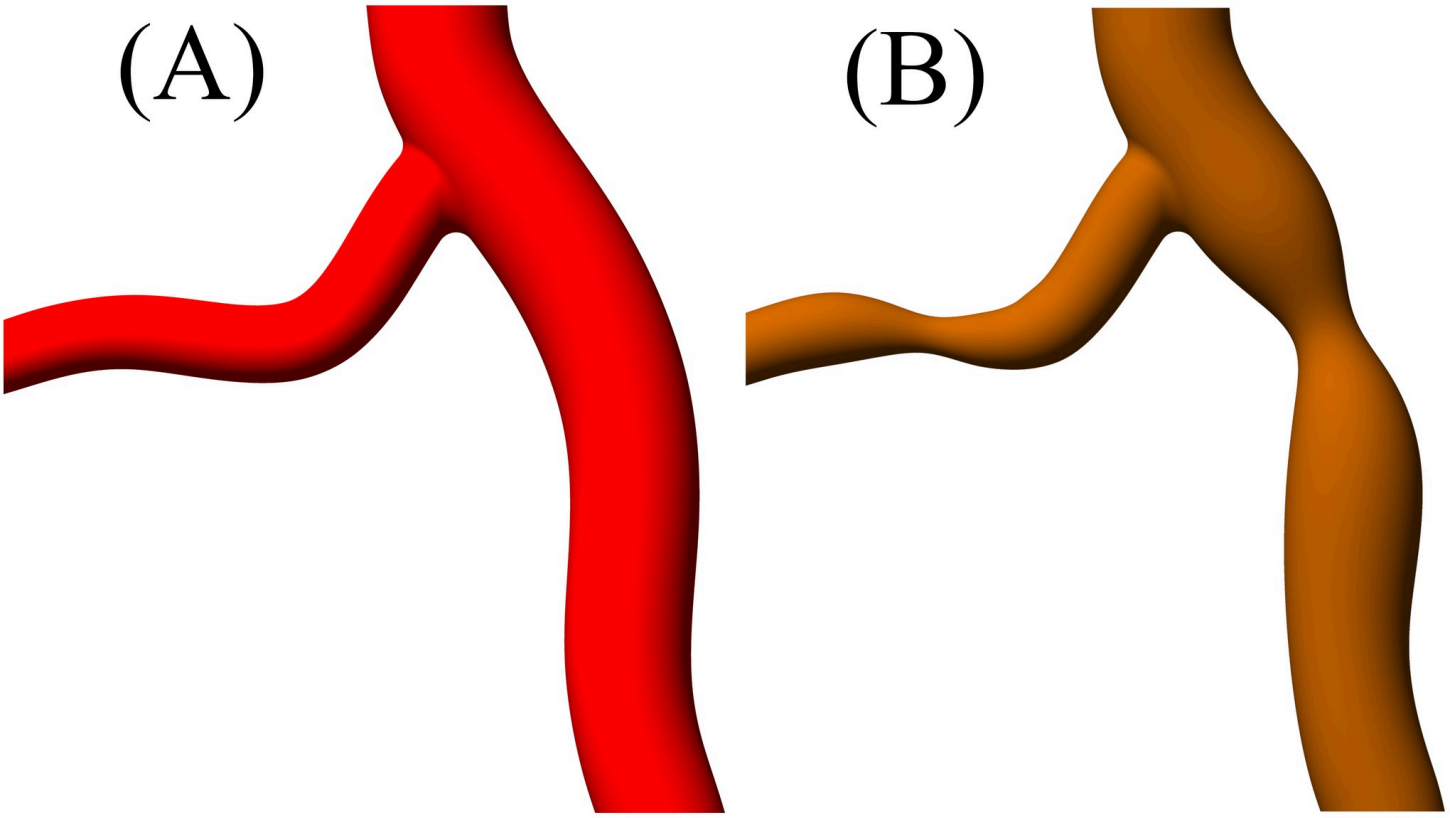
Comparison between (A) healthy and (B) diseased arterial bifurcation with stenosis.

A common coronary artery disease is the growth of atherosclerotic plaques within the lumen surface resulting in stenosis and a reduction in lumen diameter. Based upon an *in vivo* study of 140 patients, the most common occurring stenosis in coronary artery bifurcations is a continuous and diffuse plaque in both the LAD and LCx branches [33]. Stenosis can be defined as the reduction in lumen diameter due to plaque formation (Eq 1), with this study using a 50% stenosis.

$$S_{p}=\frac{D_{healthy}-D_{min}}{D_{healthy}}$$ (1)

where *S*_*p*_ is the percentage stenosis, *D*_*healthy*_ is the diameter before the plaque and *D*_*min*_ is the minimum arterial dimeter at the point of maximum stenosis. A comparison between the healthy/diseased arterial geometry is shown in Fig 2.

### Rheological models

**Fig 3:**
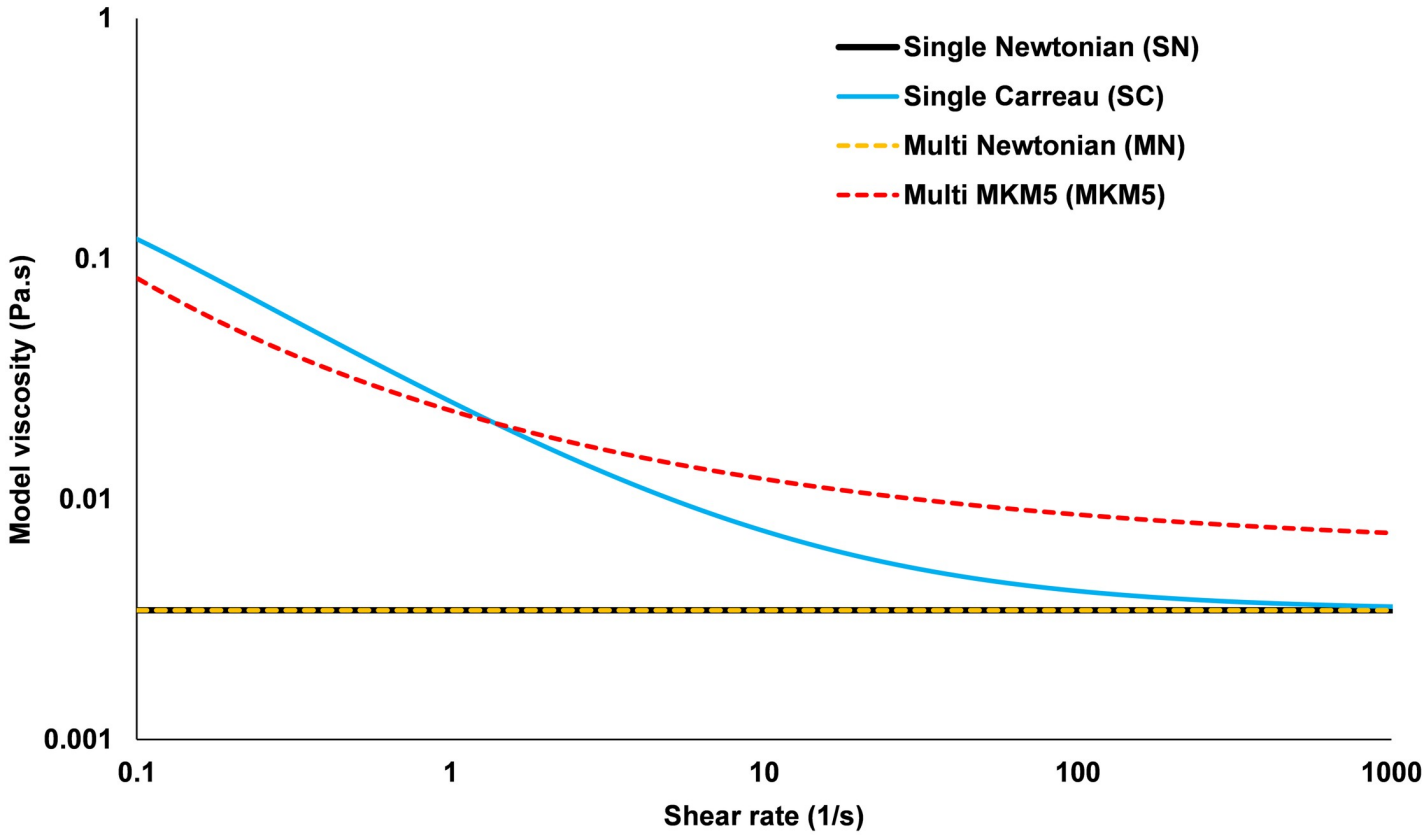
Apparent blood viscosity versus shear rate for the four rheological models (Multiphase models use a Hematocrit of 0.45).

Whole blood exhibits significant non-Newtonian properties [34], with a viscosity that depends on shear rate (Eq 2) and hematocrit (red blood cell concentration) as well as a variety of other mechanical/biological factors [16].

$$\dot{\gamma }=\sqrt{2D_{ij}∙D_{ij}}$$ (2)

where $\dot{\gamma }$ is the shear rate of the fluid, *D* is the strain rate tensor with *i*,*j* = 1,2,3 as the inner products. This study examines four different rheological models, consisting of two single-phase models (which consider blood a single homogenous fluid), and two multiphase models (which consider blood as a dilute suspension of RBC within a plasma continuum). Of the two single-phase models, one assumes blood to be Newtonian [35] with constant viscosity (Single Newtonian, SN), and the other uses a shear dependant Carreau [36] viscosity definition (Single Carreau, SC). Similarly for the two multiphase models, one uses a Newtonian approach, where the viscosity of each phase is constant (Multi Newtonian, MN) and the other uses a modified Krieger model with 5 parameters [26] (MKM5) which allows RBC viscosity to vary with both shear forces and hematocrit. The viscosity of whole blood at varying physiological shear rates for each of the four models is given in Fig 3. The accompanying mathematical definitions and coefficients for each model are provided in Table 1.

**Table 1:** Definitions for the rheological models.

Model	Viscosity definition (Pa.s)	Parameters
Single-phase Newtonian [35] (SN)	*μ* = 0.00345	-
Single-phase Carreau [36] (SC)	$\mu =\mu_{\infty }+({\mu_{0}-\mu }_{\infty }){\left[1+{\left(\lambda \dot{\gamma }\right)}^{2}\right]}^{\frac{n-1}{2}}$	*μ*_∞_ = 0.00345, *n* = 0.25
		*μ*_0_ = 0.25, *λ* = 25
Multiphase Newtonian (MN)	*μ* = *μ*_*rbc*_∅_*rbc*_ + *μ*_*p*_(1 − ∅_*rbc*_)	*μ*_*rbc*_ = 0.006163
		*μ*_*p*_ = 0.00123
Multiphase Modified Krieger Model 5 Parameters [30] (MKM5)	$\mu =\mu_{p}{\left[1-\frac{\emptyset_{rbc}}{\emptyset_{rbc,crit}}\right]}^{-n}$	*μ*_*p*_ = 0.00123
		∅_*rbc*,*crit*_ = 0.95
	$n=\{\begin{array}{cc} n_{\infty } & if\;\emptyset_{rbc}<0.2\\ n_{\infty }+n_{st} & if\;\emptyset_{rbc}>0.2 \end{array}$	*b* = 8.78084
		*c* = 2.82354
	$n_{\infty }=be^{-c\emptyset_{rbc}},\;{\;n}_{st}=\beta {\gamma^{\prime }}^{-\nu }$	*β* = 16.27775
	$\gamma^{\prime }=1+{\;(\lambda \dot{\gamma })}^{\nu_{g}}$	*ν* = 0.14275
		*λ* = 1252.64407
		*ν*_*g*_ = 2

In Table 1, *μ*_*rbc*_ is the viscosity of the RBC, *μ*_*p*_ is the viscosity of plasma, ∅_*rbc*_ is the hematocrit and ∅_*rbc*,*crit*_ is the critical hematocrit where the RBC no longer behave as a fluid. As the definitions in Table 1 are for the viscosities of whole blood, the viscous definitions required for the RBC in the MKM5 model can be inferred from a weighted average shown in Eq 3.

$$\mu_{rbc}=\frac{\mu_{blood}-(1-\emptyset_{rbc})\mu_{p}}{\emptyset_{rbc}}$$ (3)

where *μ*_*blood*_ is the definition of MKM5 viscosity provided in Table 1. This equation was also used to calculate the Newtonian viscosity of the RBC based upon a hematocrit of 0.45 and a whole blood mixture viscosity of 3.45 mPa.s.

## Numerical methods

### Governing equations

The governing equations for the continuity of mass and momentum for the single and multiphase models are given in Table 2, including additional multiphase relationships. These equations were solved numerically using the commercial finite-volume solver Fluent (Ansys v20.1, Ansys Inc., Canonsburg, PA, USA). The single-phase models assumed blood to be incompressible with a density [37] of *ρ* = 1060 kg/m^3^. The transport and phase interactions of the multiphase models were implemented using an Eulerian-Eulerian mixture model similar to other cardiovascular models [38], which considers RBC as a dilute suspension within a Newtonian plasma continuum, with a comprehensive overview of this technique available elsewhere [30].

**Table 2:** Conservation of mass and momentum for the single and multiphase models, with the volume fraction and external force (virtual mass) definitions for the multiphase models.

**Single-phase equations**	
$\frac{\partial u_{i}}{\partial x_{i}}=0$	where *u* is the velocity, *x* is the spatial coordinate, *t* is time, *μ* is fluid viscosity, *ρ* is fluid density and *p* is pressure.
$\frac{\partial u_{i}}{\partial t}+u_{j}\frac{\partial u_{i}}{\partial x_{j}}=\frac{\partial }{\partial x_{j}}(\mu \frac{\partial u_{i}}{\partial x_{j}})-\frac{1}{\rho }\frac{\partial p}{\partial x_{i}}$	
**Multiphase equations**	
$\sum_{\text{n}=1}^{2}\emptyset_{n}=1\;$	where ∅ is the volume fraction of each phase, *a*, *b* are the primary/secondary phases (plasma/RBC) respectively, *ρ*_*b*_ is the density of phase *b*, ${\vec{v}}_{b}$ is the velocity of phase *b*, p is pressure (shared by all phases), ${\bar{\bar{\tau }}}_{b}$ is the stress-strain tensor of phase *b*, *K*_*ab*_ is the interphase momentum exchange coefficient and ${\vec{F}}_{ext}\;$ are the external forces.
$\frac{\partial }{\partial t}(\emptyset_{b}\rho_{b})+\nabla ∙(\emptyset_{b}\rho_{b}{\vec{v}}_{b})=0$	
$\frac{\partial }{\partial t}(\emptyset_{b}\rho_{b}{\vec{v}}_{b})+\nabla ∙(\emptyset_{b}\rho_{b}{\vec{v}}_{b}{\vec{v}}_{b})=-\emptyset_{b}\nabla \text{p}+\nabla ∙{\bar{\bar{\tau }}}_{b}+\;\sum_{a,b=1}^{2}K_{ab}({\vec{v}}_{a}{-\vec{v}}_{b})+{\vec{F}}_{ext}$	
${\vec{F}}_{vm}=0.5\emptyset_{b}\rho_{a}(\frac{d_{a}{\vec{v}}_{a}}{dt}-\frac{d_{b}{\vec{v}}_{b}}{dt})$	

The momentum exchange coefficient between phases is derived from the RBC interfacial-area [39], and the viscous drag they experience, which is given by the Shiller-Naumann [40] model for spheres. The only external force considered was the virtual mass force, arising from the changing inertia of the plasma phase due to relative RBC acceleration. The lift due to shear is also not included due to limitations of numerical models at wide ranging Reynolds numbers/shear rates near the boundary [41] as well as only being recommended for sub-micron particles [42]. In both multiphase models, the density of plasma [17] and RBC [43] were set as *ρ*_*a*_ = 1003 kg/m^3^ and *ρ*_*b*_ = 1096 kg/m^3^, respectively. The hematocrit distribution of the domain and inlet was set at a uniform 45% based upon physiological ranges [44], however, the exact *in vivo* distribution is unknown.

### Platelet modelling

To further evaluate the hemodynamics of the coronary bifurcation, platelets were released into the domain and tracked over multiple cardiac cycles to evaluate their path lines and physical properties. The platelets were considered as 2 μm diameter rigid spheres [45] with density *ρ*_*p*_ = 1040 kg/m^3^, and were assumed not to impact the blood transport (one way interaction with fluid phases). The platelets path line is calculated by equating particle inertia to the sum of forces upon the particle from Eq 4.

$$\frac{d\vec{u_{p}}}{dt}=F_{D}(\vec{u}-\vec{u_{p}})+\vec{F}$$ (4)

where $\vec{u}$ and $\vec{u_{p}}$ are the fluid and platelet velocity, respectively, *F*_*D*_ is the drag force (Eq 5) and $\vec{F}$ is the contribution of external forces.

$$F_{D}=\frac{18\mu }{\rho_{p}{d_{p}}^{2}}\frac{C_{D}Re}{24}$$ (5)

where *d*_*p*_ is platelet diameter, *Re* is the relative Reynolds number and *C*_*D*_ is the drag coefficient of the platelet defined in Eq 6, using coefficients over a range of Reynolds numbers determined by Morsi and Alexander [46].

$$C_{D}=\alpha_{1}+\frac{\alpha_{2}}{Re}+\frac{\alpha_{3}}{{Re}^{2}}$$ (6)

Whilst the most significant force upon the platelets is viscous drag, other important forces when particle/fluid densities are similar are the pressure gradient and virtual mass forces (Eqs 7 and 8) acting upon the platelet and are given, respectively, by:

$$\vec{F_{p}}=\frac{\rho }{\rho_{p}}\vec{u_{p}}\;\nabla \;\vec{u}$$ (7)

$$\vec{F_{vmp}}=\frac{\rho }{{2\rho }_{p}}(\vec{u_{p}}\;\nabla \;\vec{u}-\frac{d\vec{u_{p}}}{dt})$$ (8)

Additional forces such as those due to buoyancy, Magnus force, and Brownian motion are not included as they were assumed negligible [47]. The time dependant shear history of the platelets was evaluated using the approach first presented by Bluestein *et al*. [48], which determines the ‘level of activation’ (LOA) via the cumulative sum of a platelets exposure time (Δ*t*) to shear stresses within the blood (Eq 9).


$$L.O.A=\sum \left[(\mu ∙\dot{\gamma })∙\Delta t\right]$$ (9)

**Fig 4:**
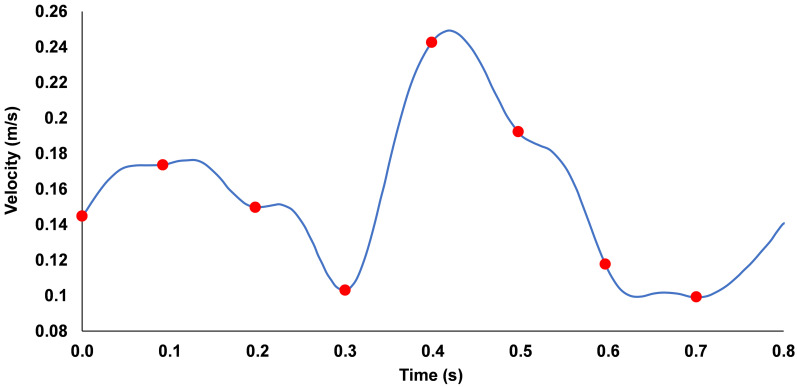
Variation of inlet velocity against time applied to the coronary artery, red dots indicate the 0.1 s intervals during which platelets were released into flow.

This relativistic measure can identify potential regions for the activation/aggregation of platelets, with an elevated LOA being defined as values >66^th^ percentile from the total range. Platelets were released in 8 separate groups from the physiological inlet at 0.1 s intervals (Fig 4), with each mesh element seeding one platelet, for a total of 13439 per release which approximates the normal human concentration [49]. The platelets were realised after 3 cardiac cycles (2.4 s) had been completed and were tracked through the flow field for a total period of 4 s (up to 6.4 s).

### Boundary conditions

#### Inlet and outlet

A typical physiological left main coronary velocity waveform [50] (Fig 4) was fitted to a Fourier series and scaled to match the model size, with a Reynolds number range from 277–691.

A parabolic velocity profile describing developed laminar flow [51] was implemented to the waveform in Fig 4 using Eq 10.

$$u(r,t)=2V(t)[1-(\frac{r^{2}}{R^{2}})]$$ (10)

where *V*(*t*) is the inlet velocity waveform, *r* is the radial position at the inlet and *R* is the maximum radius of the inlet (4.5 mm). A constant pressure of 120 mmHg (16 kPa) which approximates with mean systolic blood pressure [52] was applied at the outlets.

### Hemodynamic parameters

To compare the differences in the hemodynamics arising from the different geometries and rheology models, parameters commonly associated with the assessment of atherosclerosis are defined in Eqs 11–17.

Wall shear stress (WSS) is the force exerted by the flow of blood over the artery surface, and is a function of the viscosity and velocity gradient of the fluid, defined in Eq 11.

$$\tau_{w}=\mu {\frac{\partial u_{t}}{\partial n}|}_{wall}$$ (11)

where *τ*_*w*_ is wall shear stress, *u*_*t*_ is tangential wall velocity and *n* is unit vector perpendicular to the wall. The WSS fluctuates throughout the cardiac cycle, and so averaging WSS over the cycle can better represent the flow conditions. Time-averaged wall shear stress (TAWSS) (Eq 12) has been extensively correlated to the onset and progression of CVD such as atherosclerosis, with a magnitude <1 Pa considered atherodegenerative based upon multiple *in vivo* and *in vitro* studies [7, 53, 54].

$${\overset{-}{\tau }}_{w}=\frac{1}{T}\int_{0}^{T}|\tau_{w}|\;dt$$ (12)

where ${\overset{-}{\tau }}_{w}$ is the TAWSS and *T* is the length of one cardiac cycle. The oscillatory shear index (OSI) is a dimensionless parameter introduced by He & Ku (Eq 13), with values near 0 indicating unidirectional flow, and values near 0.5 indicating highly oscillatory flow [55]. An *in vitro* study correlated increased particulate adhesion with elevated OSI [56].

$$\theta_{i}=\frac{1}{2}(1-\frac{\left|\int_{0}^{T}\tau_{w}\;dt\right)}{\int_{0}^{T}\left|\tau_{w}\right|\;dt}|$$ (13)

where *θ*_*i*_ is the OSI. Another WSS based parameter commonly used to assess atherodegenerative conditions is the relative residence time (RRT), which provides a relative measure of the time blood spends in an arbitrary near-wall region [57], and is defined in Eq 14.

$$t_{r}=\frac{k}{(1-2\theta_{i}){\overset{-}{\tau }}_{w}}$$ (14)

where *t*_*r*_ is the RRT, and *k* is a proportionality constant arising from the near-wall assumption set as *k* = 1. Instead of simply reporting magnitudes of OSI and RRT, the percentage area of artery wall with values greater than the 66^th^ percentile is instead quantified (OSI_66_ and RRT_66_ respectively) to allow for more meaningful comparisons of the disturbed hemodynamics between geometries, as described by De Nisco *et al*. [58].

In addition to these WSS based parameters, the time-averaged non-Newtonian importance factor (TANNIF) introduced by Ballyk *et al*. [59] is used to quantify the extent of non-Newtonian flow in the artery, with values outside of unity indicating highly non-Newtonian flow (Eq 15).

$${\overset{-}{I}}_{L}=\frac{1}{T}\int_{0}^{T}\frac{\mu }{\mu_{N}}\;dt$$ (15)

where ${\overset{-}{I}}_{L}$ is the TANNIF and *μ*_*N*_ is the Newtonian viscosity listed in Table 1 (3.45 mPas). The final two parameters evaluated are based upon recent developments in correlating the vorticity induced flow disturbance to patient-specific artery models [60], comparing the increase in vorticity in healthy/diseased geometries to the type of plaques/lesions which develop. The time-average of the vorticity is taken over a cardiac cycle, and then averaged over the blood volume in the bifurcation ($\overset{-}{\omega }$), with the diseased vorticity index (DVI) being the difference in $\overset{-}{\omega }$ between the healthy/diseased cases (Eqs 16 and 17 respectively).

$$\overset{-}{\omega }=\frac{1}{V}\int_{0}^{V}\frac{1}{T}\int_{0}^{T}|\omega |dt\;dV$$ (16)

$$V_{D}={\overset{-}{\omega }}_{D}-{\overset{-}{\omega }}_{H}$$ (17)

where $\overset{-}{\omega }$ is the volume average of the time-averaged vorticity and *V*_*D*_ is the DVI.

### Solver settings

The solution to the above equations was obtained numerically via the discrete form of the SIMPLE algorithm for the pressure-velocity coupling, using a first order time discretisation scheme. All models were solved in parallel, utilising 120 cores on high performance computing nodes. To generate the hemodynamic parameters, each model was solved for a total of 3 cardiac cycles (2.4 s), with a time step of 1 ms, and all hemodynamic results being evaluated over the final cardiac cycle. The platelet model was then solved for an additional 4 s of flow time. Convergence criteria per iteration was set at a mass continuity residual <10^−4^, and velocity residuals <10^−6^ with the single and multiphase models taking an average wall clock time of 7 and 24 hours, respectively.

### Mesh convergence

**Fig 5:**
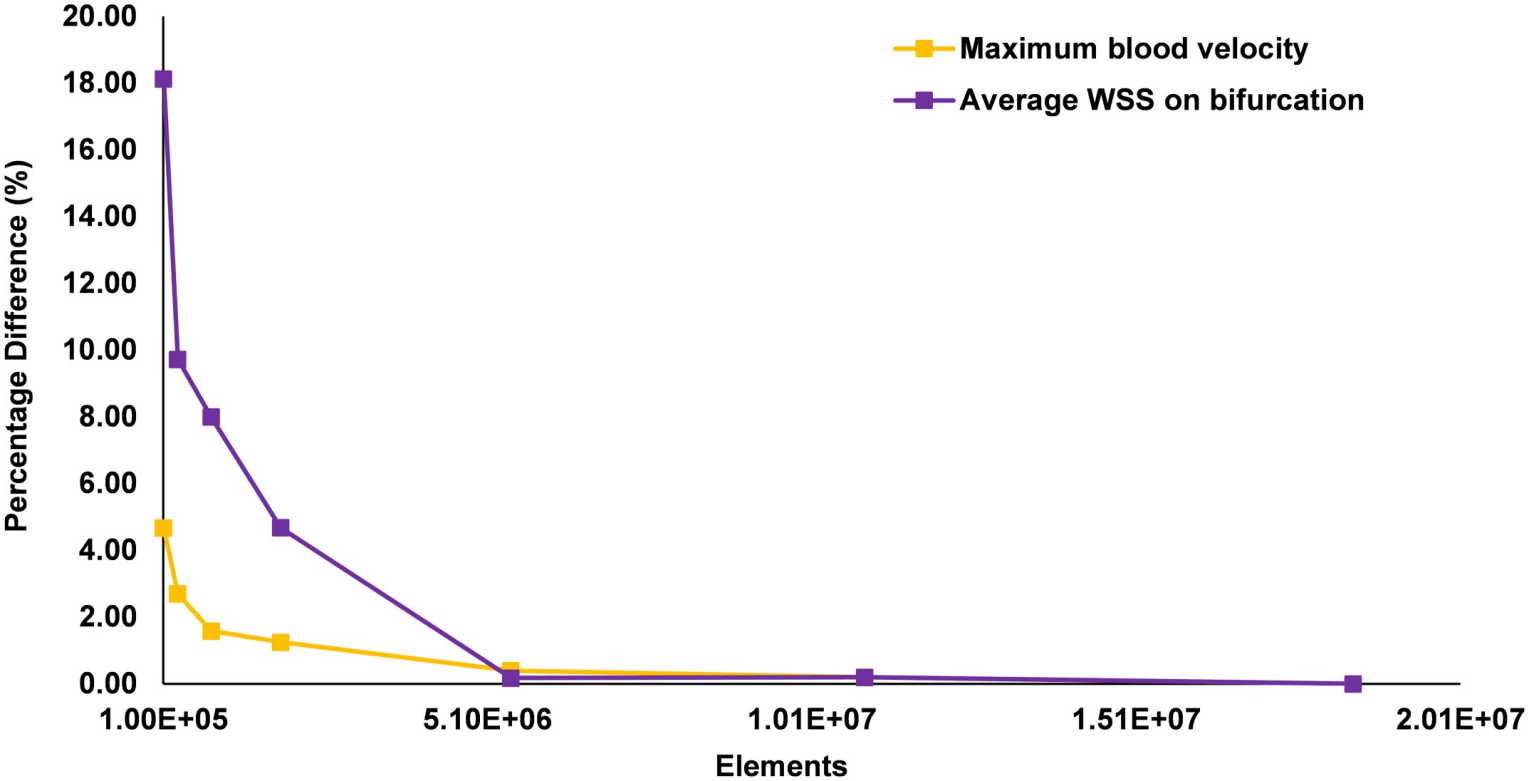
Percentage difference in peak velocity and bifurcation average wall shear stress for 7 mesh refinements.

**Fig 6:**
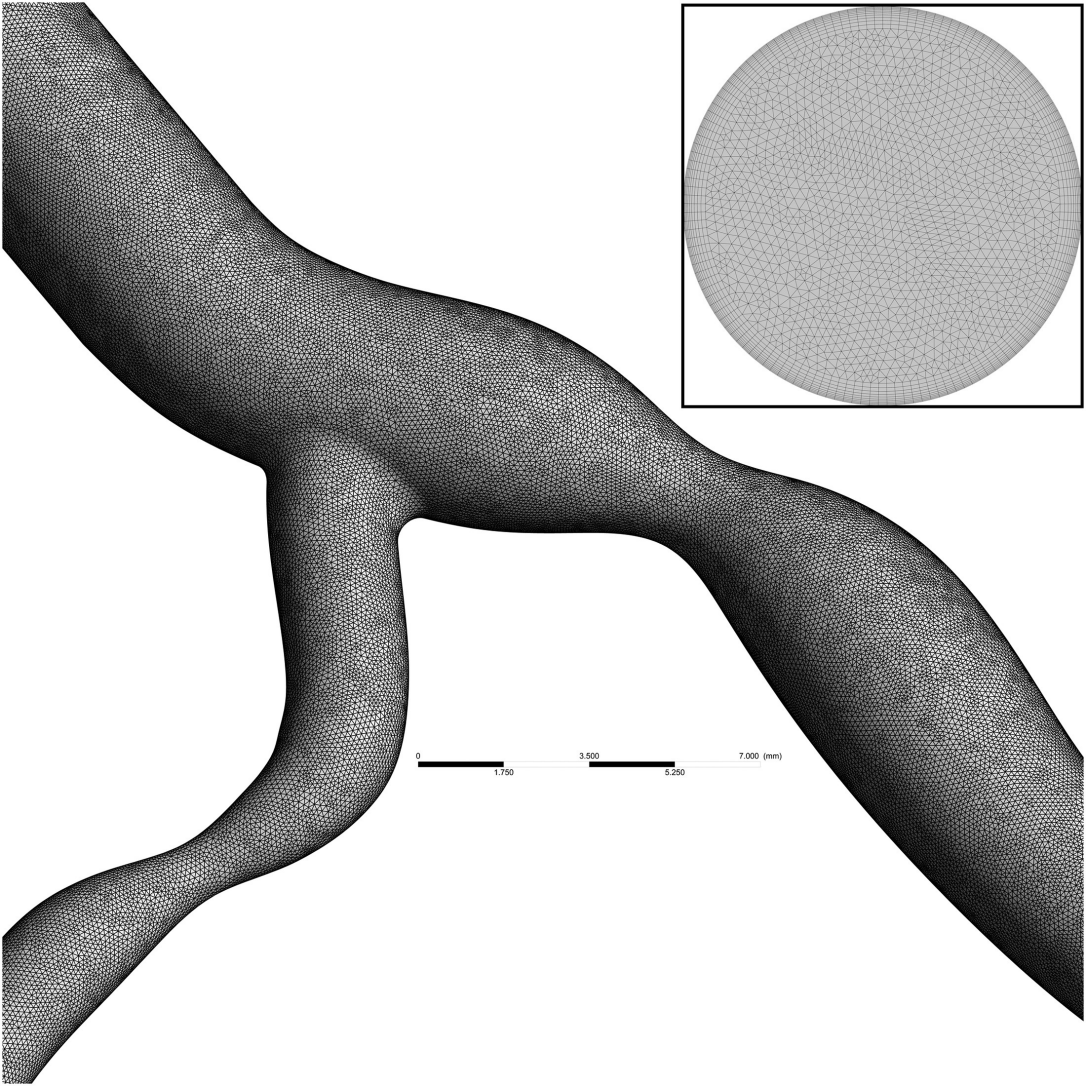
Mesh on surface of diseased coronary artery bifurcation and a cross section at inlet showing inflation layers near boundary.

To ensure a mesh independent solution is obtained, simulations of the diseased bifurcation were performed at 7 incrementally increasing mesh refinements. These were performed as a steady-state simulation, using the maximum inlet velocity of 0.5 m/s. A final meshing criterion was selected once the percentage difference of peak blood velocity and average WSS on the bifurcation flow divider were both below 0.4% for each mesh refinement. The results of the convergence study are shown in Fig 5, with the final mesh consisting of 5.4 million mostly tetrahedral elements, with a 0.25 mm thick prismatic layer of 10 elements around the lumen walls for an accurate boundary layer formulation (Fig 6). The mesh has minimum, average and maximum edge length of 0.001, 0.1 and 0.175 mm respectively, with an average element skewness and orthogonal quality of 0.20 and 0.80, respectively [61].

## Results

The hemodynamics parameters defined previously were evaluated over the 3^rd^ cardiac cycle for the healthy and diseased arteries and are reported in Table 3.

**Table 3:** Parameters averaged over the final cardiac cycle for the healthy and stenosed geometries (TA = time averaged).

Parameter	Healthy				Diseased			
	SN	SC	MN	MKM5	SN	SC	MN	MKM5
$\overset{-}{\omega }$ (1/s)	234 ± 173	232 ± 173	227 ± 196	230 ± 179	275 ± 249	271 ± 248	259 ± 256	256 ± 227
DVI (mm^3^/s)	-				4.15 x10^5^	3.90 x10^5^	3.22 x10^5^	2.58 x10^5^
TAWSS area < 1 Pa	9.48%	5.89%	5.56%	0.05%	15.09%	13.34%	10.54%	3.79%
OSI_66_	0.92%	0.87%	0.78%	0.61%	3.81%	3.62%	3.20%	2.09%
RRT_66_	5.14%	3.47%	3.31%	0.01%	11.32%	10.11%	8.23%	5.55%
Max TANNIF	1.00	3.96	1.08	2.68	1.00	13.10	1.09	6.27
Max TA Hematocrit	-		0.508	0.595	-		0.515	0.613
Min TA Hematocrit			0.321	0.204			0.282	0.167

### Shear stress parameters

**Fig 7:**
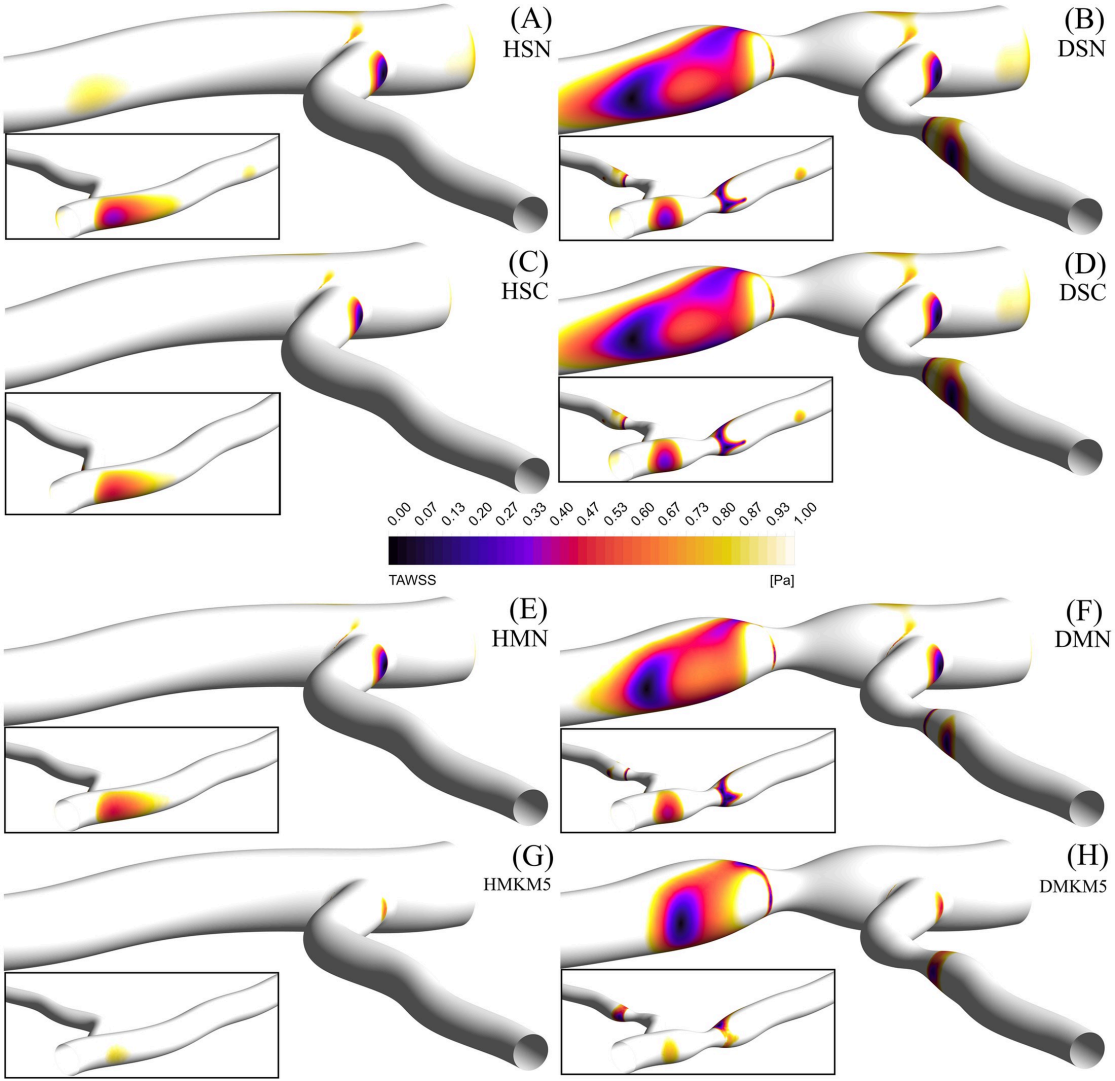
TAWSS contours of regions <1 Pa, for the left coronary bifurcation for the single Newtonian healthy (A) and diseased (B) cases, single Carreau healthy (C) and diseased (D) cases, multi Newtonian healthy (E) and diseased (F) cases, and the multi MKM5 healthy (G) and diseased (H) cases, with the reverse angle inlaid.

All models show a consistent distribution of TAWSS around the bifurcation, with regions of low TAWSS localised on the outer walls, proximal to the bifurcation and distal to the stenosis (Fig 7). In the healthy artery, all models except the MKM5 show regions with pathologically low TAWSS <1 Pa. Single-phase models show consistently greater TAWSS, OSI_66_ and RRT_66_ values compared to the multiphase models, in particular the MKM5 model. Maximum values of TAWSS occur at the throat of the stenosis, with greatest values occurring in the LCx branch in all models. Despite allowing for RBC transport and phase interactions, the MN model performs most similarly to both single-phase approaches for TAWSS results.

The introduction of stenosis results in a moderate increase in areas with a pathologically low TAWSS for all models (+5.5% average), again with the MKM5’s TAWSS results being smaller and localised closer to the stenosis (Fig 7). The SN model has the greatest results of all stress-based parameters throughout. Additionally, the TANNIF more than doubles for the SC and MKM5 models, indicating the presence of highly non-Newtonian flow in the diseased artery. Despite both the SC and MKM5 models both demonstrating non-Newtonian flow, the magnitude and distribution of TAWSS is still different, showing the importance of hematocrit on rheology and near-wall hemodynamics.

### Vorticity

**Fig 8:**
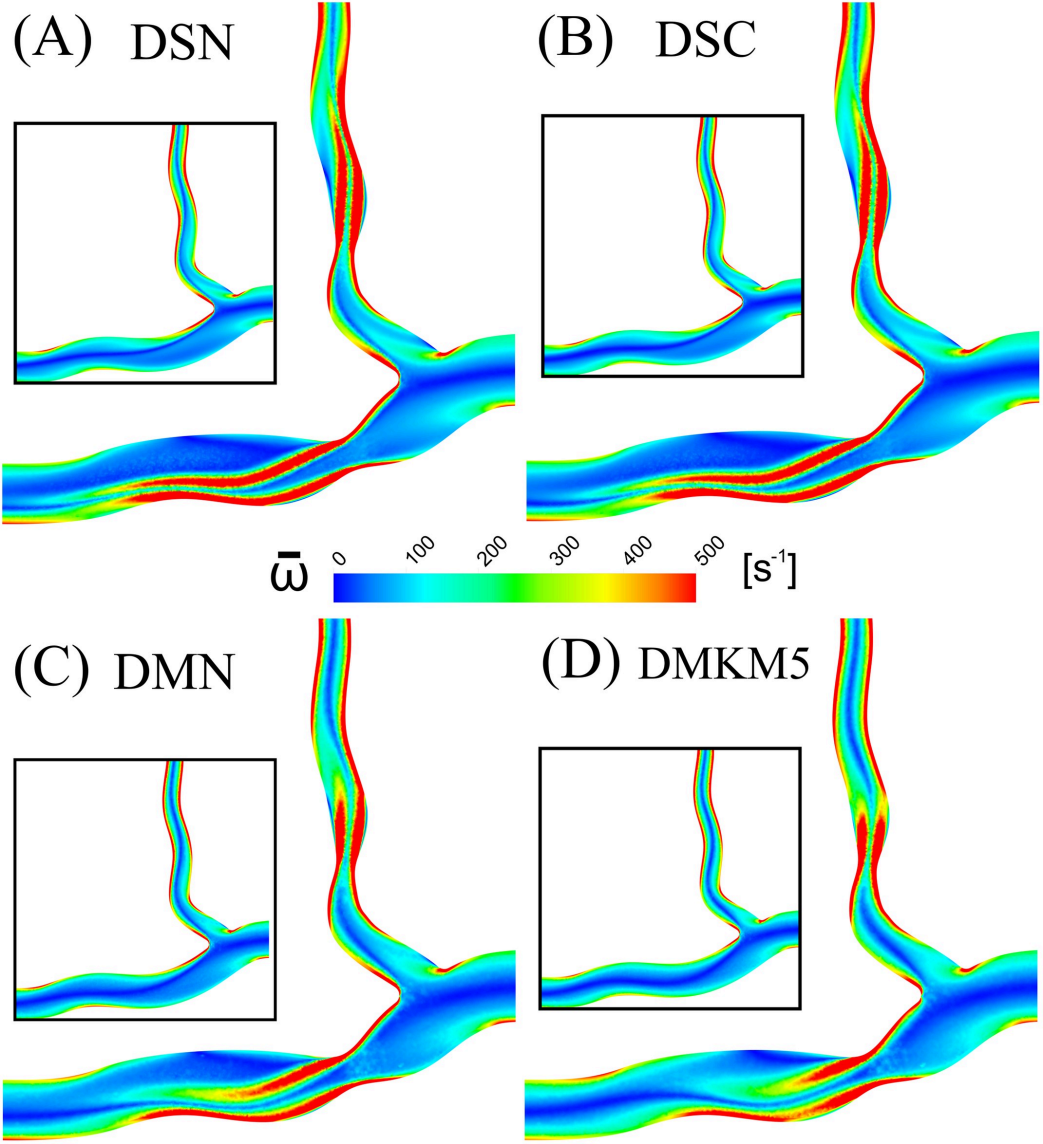
Mid-plane cross section of time averaged blood vorticity for the diseased artery: (A) Single-Newtonian, (B) Single-Carreau, (C) Multi-Newtonian and (D) Multi-MKM5 models. Results for the healthy artery are inlaid as reference.

Results in terms of vorticity were consistent across all rheological models, with the largest difference from the Newtonian model being 2.2% (MN) and 5.2% (MKM5) in healthy and diseased arteries, respectively. The regions of disturbed flow result in a wide range of vorticity values, and hence the large standard deviation in values across the cycle. The disruption to the flow caused by the stenosis increased the vorticial nature of the flow for all models, with an average increase of 15%. The reduction in lumen diameter and increased shear at the throat of the stenosis is shown to increase the vorticity of the flow distal to the throat of the stenosis, separating from the wall and spreading across the artery (Fig 8). Multiphase models predict a more localised disruption to the vorticity, closer to the throat of the stenosis, with the flow normalising much sooner than in the two single-phase approaches. Multiphase models yielded a smaller DVI than the single-phase, with the MN and MKM5 having a difference of 22.4% and 37.8% from the single-phase Newtonian, respectively.

### Hematocrit

**Fig 9:**
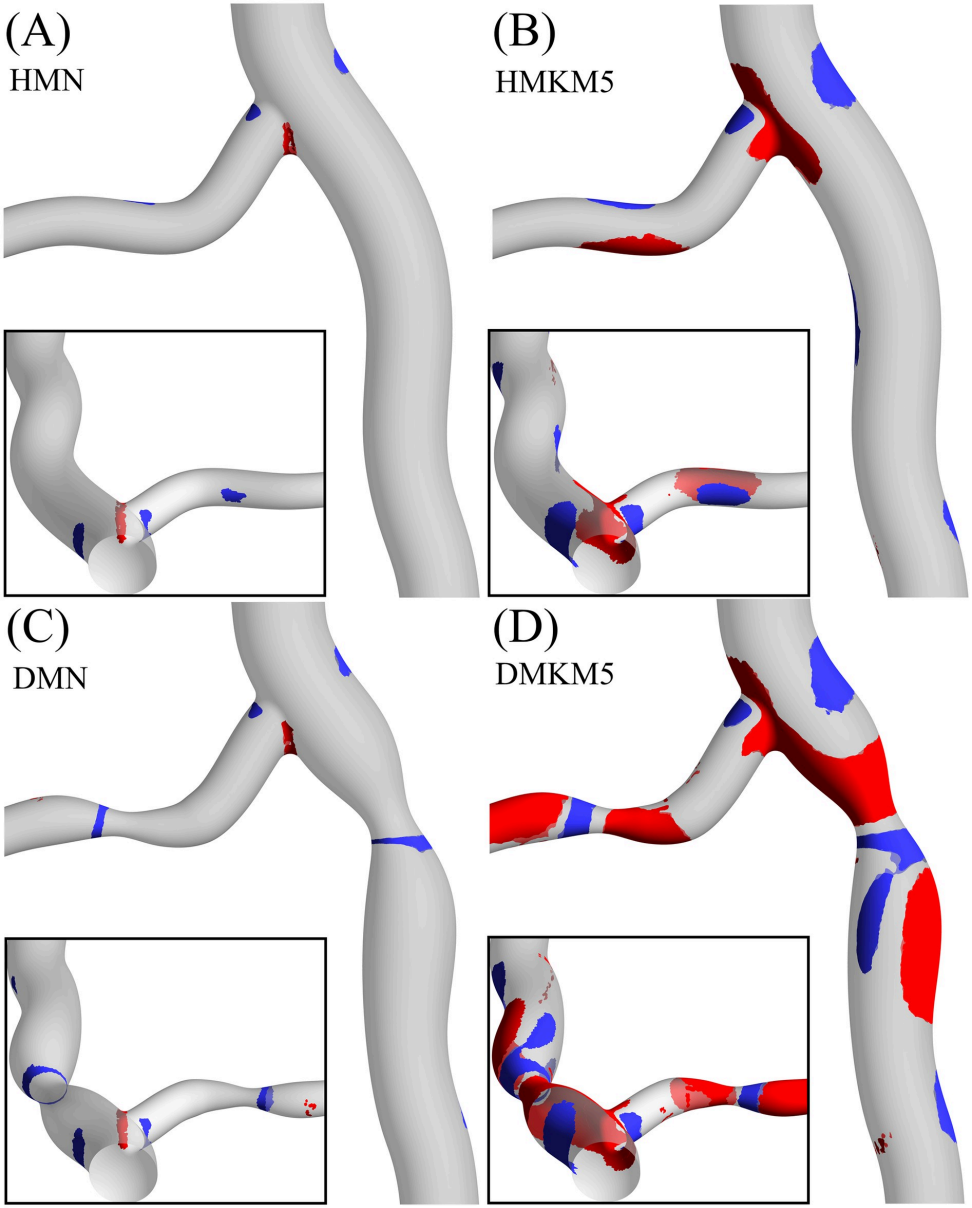
Red and blue iso-contours indicating regions of high (>0.5) and low (<0.4) TA hematocrit, respectively, for the healthy Multi-Newtonian (A) and Multi-MKM5 (B) models, and for the diseased Multi-Newtonian (C) and Multi-MKM5 (D) models, with reverse views inlaid.

To further analyse the differences between the two multiphase models, the distribution of hematocrit around the bifurcation in both the healthy and diseased cases is shown in Fig 9. Despite a uniform hematocrit inlet condition of 0.45 for both models, there is a significant difference in RBC concentration and hence phase transport between the two models. The MN model shows minimal deviation from the uniform distribution, even in the significantly disturbed flow occurring at the flow divider and the outer walls of the stenosis. The increased RBC transport arising in the MKM5 model predicts large regions of the artery with both increased and decreased hematocrit. As increased hematocrit will result in increased blood viscosity, these regions will experience elevated WSS and vice versa.

### Platelet tracking

The platelet tracking model was implemented for the multiphase MKM5 model in the healthy and stenosed arteries, with results summarised in Table 4. The Lagrangian platelet residence time data has been used to extract regions in which the RBC shear rate is sufficiently low for aggregation (<50 s^-1^) and calculate the exposure time of platelets which are continuously exposed to this low-shear environment. This exposure time was reset to 0, if a platelet experienced a shear rate >50 s^-1^ for longer than 0.1 s to account for the effects of disaggregation. Videos of platelet flow, residence time, LOA and aggregatory exposure time are available in the (S1–S3 Figs).

**Table 4:** Data from platelet tracking from the MKM5 model in the healthy/diseased artery.

Parameter	Healthy	Diseased
Platelets released across 1 cardiac cycle	106690	107512
Mean LOA (Pa.s)	2.91	3.13
Median LOA (Pa.s)	1.67	1.75
Mean residence time of all platelets (s)	1.08	1.20
Mean residence time for platelets with an elevated LOA (s)	1.47	1.53
Mean exposure time of platelets to low-shear (<50 s^-1^) aggregatory flow (s)	0.19	0.37

**Fig 10:**
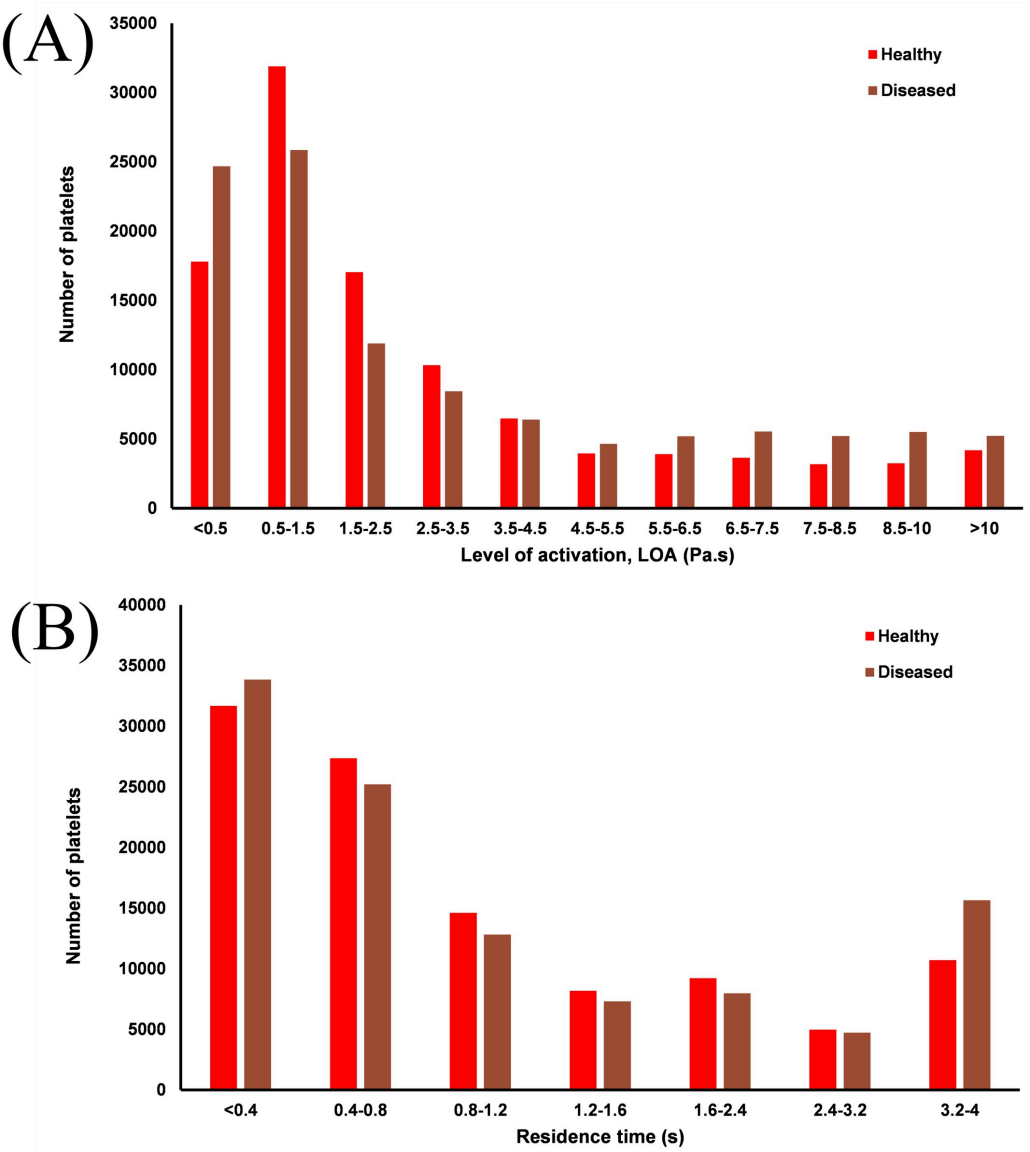
Bar charts showing distributions of LOA (A) and residence time (B) in both arteries.

The diseased artery has consistently greater levels of activation (~7%) than the healthy artery, with a large increase in the residence time of platelets which experience an elevated LOA in both the healthy (+36%) and diseased (+28%) arteries. The distribution of LOA and residence time across all platelets released can be seen in Fig 10.

In both arteries approximately 55% of platelets have residence times below 1 cardiac cycle, with platelets seeded in closest to the wall experiencing both the greatest residence times and LOA. Whilst platelets in the near-wall region have long RT in both arteries, the greater shear stress in the diseased artery results in consistently high LOA as seen in Fig 10a.

**Fig 11:**
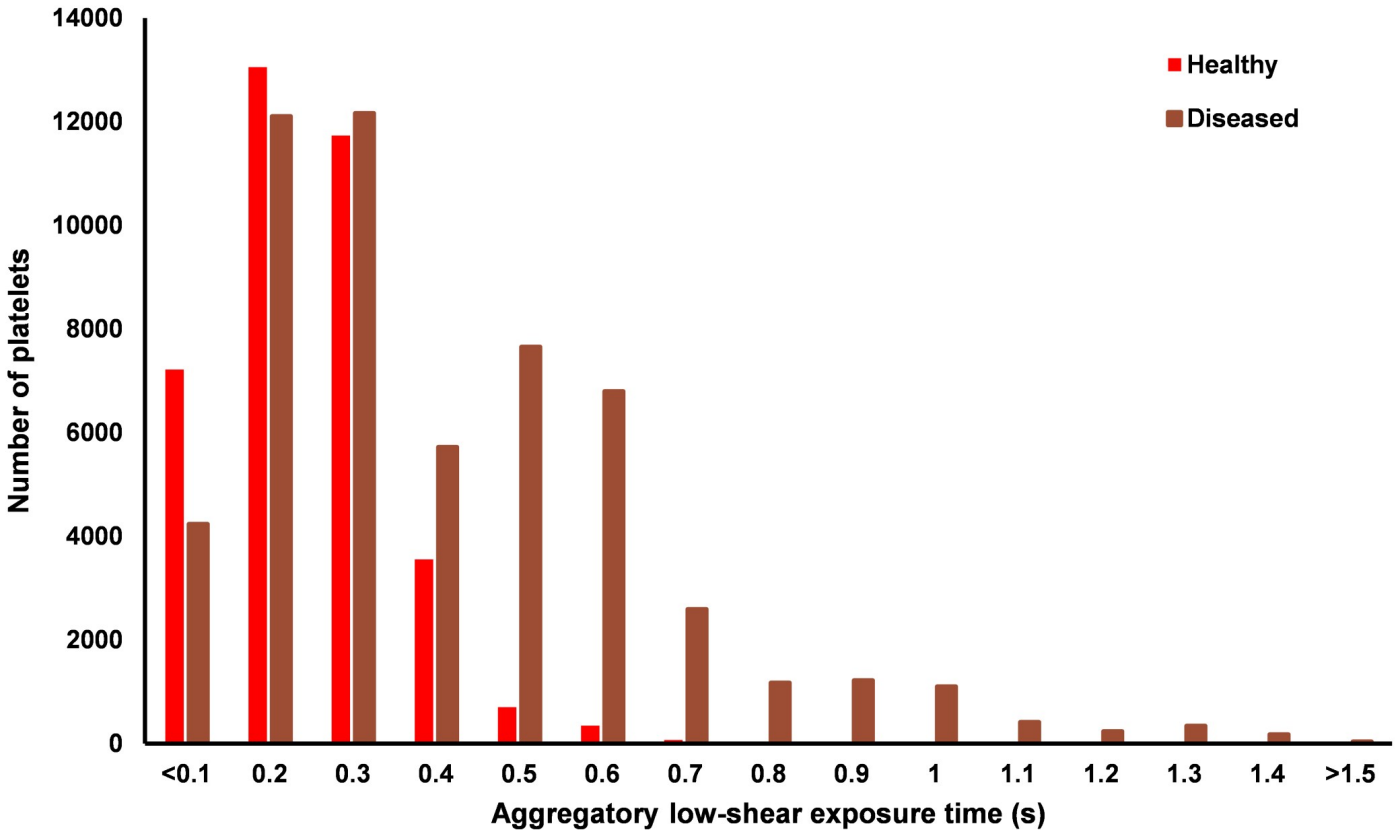
Bar chart showing the distribution of exposure time for the platelets which experienced a RBC shear rate <50 s^-1^.

**Fig 12:**
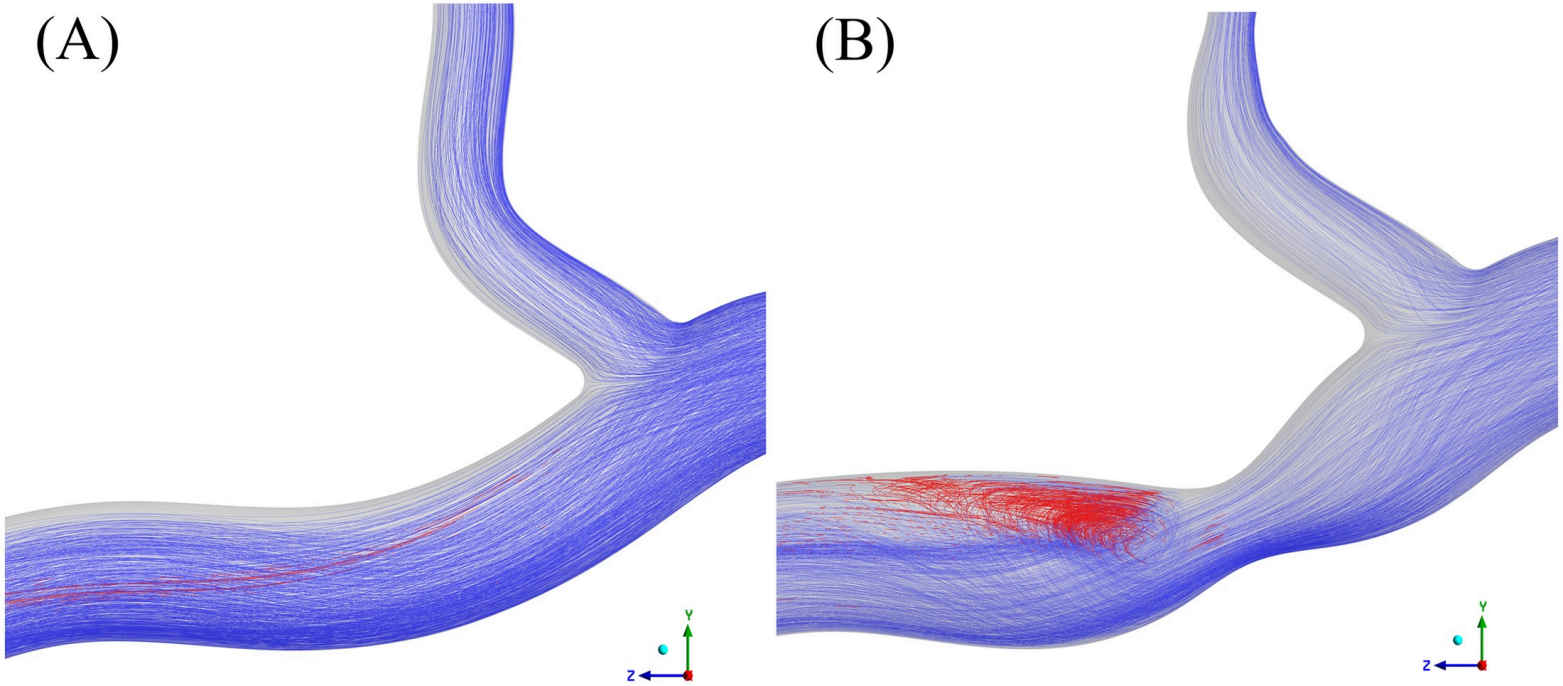
Platelet path lines for the healthy (A) and diseased (B) arteries, red indicates a low enough RBC shear rate for aggregation (<50 s^-1^).

To evaluate the aggregatory potential of the low-shear environment around the bifurcation, the distributions of exposure time to sufficiently low RBC shear rates is shown in Fig 11. There is a distinct difference between the two arteries, with no platelets in the healthy artery exposed to aggregatory shear rates for greater than 0.7 s, compared to the maximum exposure of 2.9 s in the diseased artery occurring distal to the stenosis (Videos in S1–S3 Figs). The stenosis greatly increases the area of low-shear regions in the bifurcation which in turn increase the residence time of platelets and potentially RBC. The increased aggregatory potential in the diseased artery is highlighted in Fig 12B, showing localised recirculation of platelets in an ultra-low shear, high viscosity, high hematocrit, high residence time environment distal to the throat of the stenosis.

## Discussion

This study presents for the first time, a Lagrangian platelet tracking model to assess the likely regions of platelet activation and hence indicate potential regions with an increased risk of clotting/thrombus formation alongside a comprehensive examination of the relation between blood rheology RBC aggregation/concentration and hemodynamics. Simulations of a porcine LAD/LCx stenotic bifurcation have shown the importance of non-Newtonian and multiphase rheological properties on accurate predictions of parameters associated with atherosclerosis, with initial insights into how shear stress activation of platelets catalyses atherothrombosis.

### Rheology and WSS

The links between WSS and atherosclerosis in coronary arteries have been well documented both *in vivo* and *in silico*, with regions of low TAWSS being associated with: plaque growth [62], reduced lumen diameter [53] and increased thrombus formation [63]. An *in vitro* study of coronary artery bifurcations of comparable branch angles also showed significant areas of low-shear and stagnation on the outer walls of the bifurcation [64] as seen in Fig 7. Additionally, an *in vivo* study of 506 patients with acute coronary syndrome confirmed the TAWSS patterns in Fig 7, with peak stresses occurring at the throat of stenosis, and increased regions of low TAWSS occurring 3–6 mm distal of the stenosis [53]. The increased RBC transport to these regions of stagnation distal to expansion [65] (Fig 9) is also associated with increased residence time of platelets (Fig 11) and atherogenic proteins which may further impair healthy endothelial function [66].

Of the four rheological approaches used in this study, three are based upon the viscometer data from Chien *et al*. [67], with the MKM5 model being fitted to the Brooks *et al*. [17] dataset. Discrepancies in the range of shear rates evaluated, hematocrit concentrations and temperature of blood combined with inherent limitations in measuring viscosity at the lowest shear rates [17] results in significant differences in predictions of blood viscosity across each model (Table 3). This significantly lower viscosity results in greater regions predicted to have a pathologically low TAWSS, with even the healthy bifurcation exhibiting large regions <1 Pa. Furthermore, single-phase models will never be able to predict the non-Newtonian effects arising from RBC aggregation which is a key factor in non-Newtonian descriptors. Additionally in the diseased case, whilst distributions of TAWSS are similar across all models, the atherodegenerative regions are localised much closer to the stenosis (<6 mm) as demonstrated in Stone *et al*.*’s in vivo* study [53].

### Vorticity

The vorticial nature of blood flow has been shown to impact cardiac function through multiple *in vivo* studies, with increased arterial vorticity arising in patients with right ventricular dysfunction [68]. Despite this link, coronary vorticity is thoroughly unexplored despite its potential as a non-invasive biomarker for assessing hemodynamic function [69]. The present study has demonstrated that the introduction of stenosis significantly increases the vorticity of coronary blood flow by 11–18% (across all models), with regions of high vorticity extending directly from the throat of the stenosis into the low-shear environments associated with atherosclerosis [70]. With DVI being correlated to the formation and geometry of atherosclerotic legions [60], the 38% lower value predicted by the MKM5 model compared to the SN model clearly shows the significance that RBC/plasma interactions have on flow disturbance. Based on Chu *et al*.*’s* patient-specific CFD study of coronary arteries [60], a lower DVI value may imply the growth of a more focal and severe lesion which correlates to the more localised TAWSS distribution of the MKM5 model immediately distal to the stenosis compared to the elongated SN distribution (Fig 7). Given these results and the variability in DVI with different rheology and phase models, it is therefore important to choose a sufficiently complex rheology/phase model which allows for a more accurate study of the severity of stenosis in coronary arteries.

### Hematocrit and platelets

Despite both multiphase models utilising the same velocity and hematocrit boundary conditions, predictions in RBC transport and hematocrit vary extensively in both the healthy and stenosed arteries (Fig 9). The non-Newtonian behaviour of RBC in the MKM5 model is most significant in the near-wall region due to the wide range of shear rates and hematocrit occurring near the surface [30]. These fluctuations in hematocrit close to the surface will not only impact WSS magnitudes due to increased viscosity, but also affect platelet transport [71] which is strongly linked to interaction/collisions with RBC [72], hence the limited RBC transport of the multiphase Newtonian model is a significant limitation in accurately modelling blood rheology.

Evaluating the behaviour of platelets and their association with thrombus/clot formation is relevant for vascular pathologies [14, 73] (e.g. stenosis/aneurysms) but also the function of medical devices [36, 74] (e.g. catheters/stents). Platelet transport/activation is a complex process influenced by both mechanical factors such as shear stress [75], but also through biochemical reactions with proteins/agonists within the blood itself [76], with reviews of computational approaches by Anand & Rajagopal [77] and Yesudasan *et al*. [78]. The majority of platelet models focus on predicting thrombus formation and platelet behaviour through these biochemical interactions. However, the majority of these models focus on micro-scale flow [79, 80] which are poorly suited for the larger scale hemodynamics of this coronary bifurcation. Whilst the platelet model presented in this study lacks the biochemical activation potential of other models, its key benefit is linking the level of activation to prolonged residence times in specific ‘at-risk’ areas, to indicate likely regions for aggregation, sedimentation and clot formation in diseased arteries.

The seeding of platelets in the near-wall region shows platelets lingering around the bifurcation for multiple cardiac cycles in both arteries (S1–S3 Figs). Importantly the elevated blood shear stress at the throat of the stenosis and subsequent distal low-shear stagnation increases the LOA and creates the ideal environment for adhesion and clot growth. Platelets have long been associated with atherosclerosis [81], with *in vivo* studies showing their role in both the onset [10] and progression [6] of plaque growth. Additionally, the non-Newtonian effects of RBC aggregation are shown to be relevant in the diseased artery, with exposure time to sufficiently low-shear rates exceeding the approximate time (>1.5 s) for rouleuax formation to occur [21]. The resulting increase in the viscosity of blood will lead to further aggregation and sedimentation of erythrocytes distal to the stenosis, and is both symptomatic [82] and catalytic to the deterioration of the endothelium [83] and the development of atherothrombosis [84].

### Limitations

The presented study was designed for the assessment of disease and rheology, however, there are several limitations/assumptions which impact the physiological accuracy. The principal limitations are from the geometry as it lacks some of the additional complexity and individuality arising from patient-specific scans [28, 85], including the vessel torsion which is one factor in the development of atheroprotective helical flow [86]. As applying boundary conditions of helical flow is not well documented, and whilst torsion is not directly associated with low WSS [58], the lack of helical flow development may impact hemodynamic predictions. Furthermore, only the main bifurcation branch was considered (LAD-LCx) as it is one of the most common locations for plaque growth [33], however, neglecting the additional downstream branches may alter pressure gradients and hence result in overestimation of WSS [87]. Despite this, the comparisons between rheological models are unbiased and unaffected.

Additional differences in flow boundary conditions arising from the implantation of stenosis could not be implemented in the model as adjusting the inlet condition without a suitable patient-specific profile would only further introduce errors/randomness and hence may limit differences between the two models.

The Lagrangian method of platelet simulation which determined activation only due to time-averaged shear stresses neglects the contribution of biochemical interactions in platelet activation. This discrete approach was chosen due to the macro-scale focus of this geometry and the inability of micro-scale (including majority of biochemical approaches) models to assess the trajectory, residence time and shear history of individual platelets.

Due to the sheer number of RBC in full sized arteries [22], individual RBC are not simulated in a Eulerian-Eulerian model, and so the effects of RBC deformation on their rheology/sedimentation cannot fully be included. Despite this, the momentum exchange coefficient (*K*_*ab*_) between RBC/plasma is a function of Reynold’s number/drag coefficient which will partially account for changes in shape due to flow conditions. With this study indicating aggregatory potential even in large arteries, we hope to expand current multiphase, macroscale rheology models to incorporate these aggregatory behaviours by linking factors such as: residence time, low-shear exposure and hematocrit to experimental rheometer measurements of RBC aggregates.

## Conclusion

In this study, the choice of rheological model has been shown to strongly influence the assessment of coronary hemodynamic parameters. Single-phase models oversimplify crucial low-shear blood characteristics compared to the non-Newtonian multiphase model (MKM5) which provides a better representation of *in vivo* measurements. Whilst single-phase or Newtonian models may be acceptable for representing physiological undisturbed flow, the increased exposure time (>1.5 s) to aggregatory levels of low-shear in diseased arteries will impact crucial near-wall hemodynamics. Moreover, the wide ranges of hematocrit (0.163–0.617) and elevated vorticity (+15%) occurring distal to stenosis could impair the healthy function of the endothelium via pathological regions of TAWSS and the disruption of advection/diffusion of proteins/nutrients due to altered rheological properties. Additionally, the thrombogenic potential of stenotic arteries has been further classified, with platelets located in the near-wall region experiencing the highest LOA, and subsequently experiencing lengthier residence times (+32% increase) in the low-shear, stagnant region distal to the stenosis.

## Supporting information

S1 Fig Animation of platelet flow showing exposure to sufficiently low aggregatory shear levels (<50 s^-1^) in the diseased MKM5 coronary artery bifurcation. (MP4)

S2 Fig Animation of platelet flow showing the residence time of platelets in the diseased MKM5 coronary artery bifurcation. (MP4)

S3 Fig Animation of platelet flow showing level of shear stress in the diseased MKM5 coronary artery bifurcation. (MP4)
